# Automated measurement of cell motility and proliferation

**DOI:** 10.1186/1471-2121-6-19

**Published:** 2005-04-14

**Authors:** Alfred Bahnson, Charalambos Athanassiou, Douglas Koebler, Lei Qian, Tongying Shun, Donna Shields, Hui Yu, Hong Wang, Julie Goff, Tao Cheng, Raymond Houck, Lex Cowsert

**Affiliations:** 1 Automated Cell, Inc. 390 William Pitt Way, Pittsburgh, PA, 15238 USA; 2 University of Pittsburgh Cancer Institute, Research Pavilion at The Hillman Cancer Center, 5117 Center Ave, Pittsburgh, PA, 15213-1863 USA

## Abstract

**Background:**

Time-lapse microscopic imaging provides a powerful approach for following changes in cell phenotype over time. Visible responses of whole cells can yield insight into functional changes that underlie physiological processes in health and disease. For example, features of cell motility accompany molecular changes that are central to the immune response, to carcinogenesis and metastasis, to wound healing and tissue regeneration, and to the myriad developmental processes that generate an organism. Previously reported image processing methods for motility analysis required custom viewing devices and manual interactions that may introduce bias, that slow throughput, and that constrain the scope of experiments in terms of the number of treatment variables, time period of observation, replication and statistical options. Here we describe a fully automated system in which images are acquired 24/7 from 384 well plates and are automatically processed to yield high-content motility and morphological data.

**Results:**

We have applied this technology to study the effects of different extracellular matrix compounds on human osteoblast-like cell lines to explore functional changes that may underlie processes involved in bone formation and maintenance. We show dose-response and kinetic data for induction of increased motility by laminin and collagen type I without significant effects on growth rate. Differential motility response was evident within 4 hours of plating cells; long-term responses differed depending upon cell type and surface coating. Average velocities were increased approximately 0.1 um/min by ten-fold increases in laminin coating concentration in some cases. Comparison with manual tracking demonstrated the accuracy of the automated method and highlighted the comparative imprecision of human tracking for analysis of cell motility data. Quality statistics are reported that associate with stage noise, interference by non-cell objects, and uncertainty in the outlining and positioning of cells by automated image analysis. Exponential growth, as monitored by total cell area, did not linearly correlate with absolute cell number, but proved valuable for selection of reliable tracking data and for disclosing between-experiment variations in cell growth.

**Conclusion:**

These results demonstrate the applicability of a system that uses fully automated image acquisition and analysis to study cell motility and growth. Cellular motility response is determined in an unbiased and comparatively high throughput manner. Abundant ancillary data provide opportunities for uniform filtering according to criteria that select for biological relevance and for providing insight into features of system performance. Data quality measures have been developed that can serve as a basis for the design and quality control of experiments that are facilitated by automation and the 384 well plate format. This system is applicable to large-scale studies such as drug screening and research into effects of complex combinations of factors and matrices on cell phenotype.

## Background

Cell-matrix interactions are key components of many physiological processes in health and disease. Frequently these interactions result in changes in cellular motility, morphology, and/or growth, and so quantitation of these changes is useful for comparing matrix and soluble factor effects and for assessing sensitivity of cells to varying concentrations of these factors [1,2]. A variety of methods are used to measure cell migration, including most commonly the transwell assay [3] often modified by fluorescence quantitation [4], and less commonly the under-agarose migration assay [5], the soft-agarose drop method [6], the phagokinetic track motility assay for phagocytic cell types [7], wound healing [8], and time-lapse video microscopy.

Although the transwell assay has been applied to random migration [9], video time-lapse microscopy provides advantages by yielding actual speeds of individual cells and additional features of motion, e.g. persistence [10]. The video time-lapse approach has been applied since the late 1930's using film-projected images and manual methods for tracking cell paths for determination of velocities [11]. The introduction of video imaging and computer-assisted methods of tracking have aided this approach [12,13]. However, even with computer-assisted methods, analysis of video time-lapse images can be labour intensive, particularly if the data have been gathered over extended time periods, and the opportunities for human fatigue and inadvertent selection using such methods may introduce bias. Moreover, the normal cell culture environment must be maintained during imaging. Although sophisticated studies are being conducted within special chambers [14] or under mineral oil [15], an ideal system would incorporate acquisition of images simultaneously from multiple wells under normal culture conditions maintained throughout the entire experiment.

A fully automated system for acquiring and analysing time-lapse images over extended time periods from multiple wells within 384 well plates has been developed [16-21]. The system includes an electronically controlled incubated cell culture environment for continuous monitoring over extended periods. Automated image analysis is used to determine cell morphological properties and cell location, and proprietary algorithms are used to construct cell paths or tracks through time, yielding magnitude and direction of motion. Cell proliferation is also monitored based upon processing of the same set of images. The data set provides a rich source of unbiased quantitative information about cell behaviour that can be accessed at the individual cell level and filtered in a manner similar to gating in flow cytometry. Here we apply this technology to examine responses of osteoblast-type cells to surface coatings of extracellular matrix compounds that may be involved in osteoblast differentiation and growth. This study is part of an ongoing effort seeking to uncover underlying factors that influence osteoblasts in the process of osteogenesis and in the dysregulation of this process leading to osteoporosis.

## Results

### Differential cell velocity on different ECM coatings

KM101 cells and MG-63 cells were chosen as models for this study because of their potential for differentiation into cells with osteoblast-like phenotypes. KM101 cells, from primary human bone marrow stroma [22], have been shown to secrete bone-type alkaline phosphatase upon differentiation [Julie Goff, personal observations]. The MG-63 cell line, originating from a human osteosarcoma [23], exhibits characteristics of bone forming cells including high levels of 1,25-(OH)_2_D_3_-responsive alkaline phosphatase activity and osteoblast-like regulated synthesis of osteocalcin and collagen type I [24]. Responses in cell motility may reflect functional changes that accompany osteoblast migration into areas of newly forming bone.

**Figure 1:**
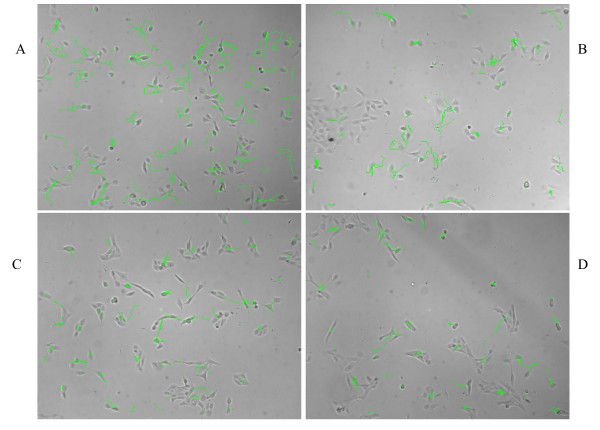
**Example tracks from KM101 cells on different surface coatings**. Single time-point images shown were acquired at 36 hours elapsed time from KM101 cells seeded into a 384 well plate pre-treated with 10 ug/ml solutions of (A) laminin, (B) collagen I, (C) collagen IV, (D) and mock coated. Tracks (green lines) have been superimposed on the images to indicate the paths of cells or clusters of cells over the previous set of 15 images (7.5 hours). Track continuity is broken in cases where two objects collide, and tracks are reinitiated upon object separation. Longer tracks reflect uninterrupted tracks and higher cell velocities. Within a track, each straight-line segment represents the distance between object centroid positions in successive images taken 30 minutes apart.

For KM101 cells and to a lesser extent for MG-63 cells, increased migratory activity on laminin coated surfaces in comparison with the other extracellular matrix compounds, collagen type I, collagen type IV, and mock-coated plastic, is evident when viewing time lapse video sequences [see Additional file 1]. Individual cell tracks were automatically constructed by the software algorithms (see Methods), and the tracks are seen to be longer on laminin I coated surfaces than on collagen type I, collagen type IV, or mock-coated plastic for KM101 cells over the same time period (Figure 1). Track length over equal time intervals correlates with velocity, and so these images demonstrate faster migration of KM101 cells on laminin-coated surfaces. It is not clearly evident from visual inspection whether less-pronounced effects are occurring in either cell type with the other surface coatings, and to what extent, if any, the effects are changing over time.

### Data selection for single live-cell velocity

**Figure 2:**
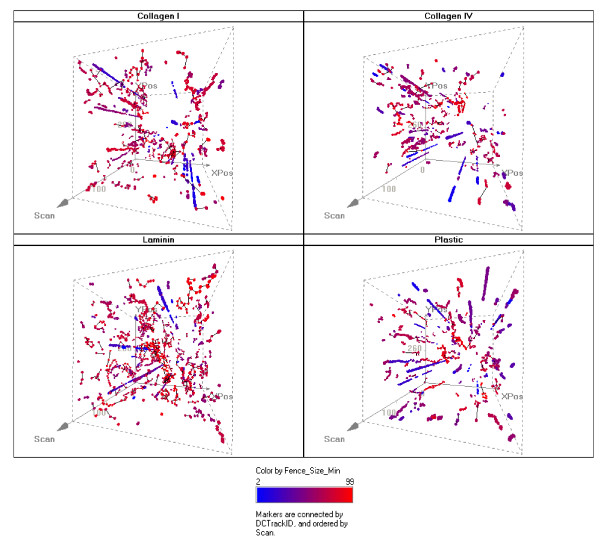
**3D visualization of tracks over time**. The 2 dimensional culture surface from within an imaged well is represented in the plane of the paper with the time dimension (labelled as "scan") represented by the third dimension coming up toward the reader. More distant time is perceived to be deeper beneath the surface of the paper using exaggerated perspective. Cell object positions appear at each scan where objects met the combined criteria of having an area less than 3000 sq microns and a track length of at least 3 segments. Position markers are colored according to fence size with blue indicating smaller, and red indicating larger fence size. Stationary objects are readily recognized by persistently straight "vertical" paths toward the reader through time, because their x, y coordinates changed very little. A total elapsed time of approximately 75 hours is represented from the "bottom" to the "top" of the 3D representation. Four different surface coatings are shown as indicated. This display was created using Spotfire visualization software (), an important tool for accessing, analysing and reporting on the multi-parametric data from the automated system.

Automated multi-well image acquisition and analysis provides the potential to examine biological response for multiple cell types, compounds, and doses simultaneously and continuously over extended periods of time, but the data must be judiciously selected. From each image, multiple objects were segmented and tracked, and a set of filtering criteria was uniformly applied from the outset, based upon logical principles (see Methods). In addition, most view-fields included surface imperfections or adherent particles that were segmented as objects, some with areas within the range of single cells. Such objects are easily ignored and often go unnoticed when using manually assisted methods of analysis, but they present challenges for automated image processing. Stationary objects became particularly apparent when object positions were plotted over time in 3 dimensions (Figure 2). Tracks from stationary objects form nearly straight lines along the time dimension, whereas live cell tracks exhibit more arbitrary excursion in the x,y plane. It is also evident from this figure that stationary objects were often segmented and tracked throughout the extended time period of the experiment, and their cumulative contribution to the data was therefore more significant than was apparent from a 2D view of accumulated tracks such as that shown in Figure 1.

**Figure 3:**
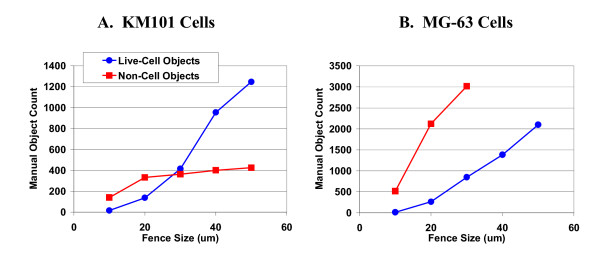
**Fence size and live-cell versus non-cell objects**. Individual track segments were manually scored from image sequences according to whether they originated from live-cell or non-cell objects. A custom viewing device permitted selection of a maximum fence size for tracks to be included in the display; in addition, only segments from tracks connecting 4 or more points were displayed. With each increasing step in fence size, the entire image sequence consisting of more than 100 images was re-scored. This process was repeated for 4 representative wells for each cell type. The cumulative total counts for live-cell (blue) and non-cell (pink) objects are plotted versus fence size on the horizontal axis for both cell types, KM101 (left) and MG-63 (right). Cell debris appeared to be more abundant from MG-63 cells after plating, but the wider range of permissible area for MG-63 cells would be expected to include also more non-cell objects of larger area. Objects of larger area would be expected to have greater segmentation and centroid position variability (see Segmentation/Outline Error) resulting in greater associated fence size. For MG-63 cells, the counting of non-cell objects was discontinued at fence sizes of 40 and above because it became simply too demanding to distinguish and count both live and non-cell objects.

For a given track, the maximum excursion in either the x or y direction is called the "fence size", and an additional filter based upon fence size was applied to exclude data from stationary objects. In order to establish fence size filter levels and to verify minimum loss of live-cell data, we devised a program to manually review and score track segments according to whether they originated from live cells or from stationary non-cell objects. For both cell types, fence sizes of 20 microns or less appeared to include the majority of non-cell objects without appreciable inclusion of live cells, and so filtering was conservatively applied at this level (Figure 3). The larger MG-63 cell area limit is associated with greater segmentation error that is in turn associated with larger fence sizes; thus the 20 micron filtering criterion was more of a compromise for slowly moving MG-63 cells than for KM101 cells. Having implemented these data filtering measures, we present overall data summaries from three experiments. It should be mentioned that this approach would not be valid in cases where significant numbers of live cells remained stationary on the culture surface.

### Kinetics of velocity response

**Figure 4:**
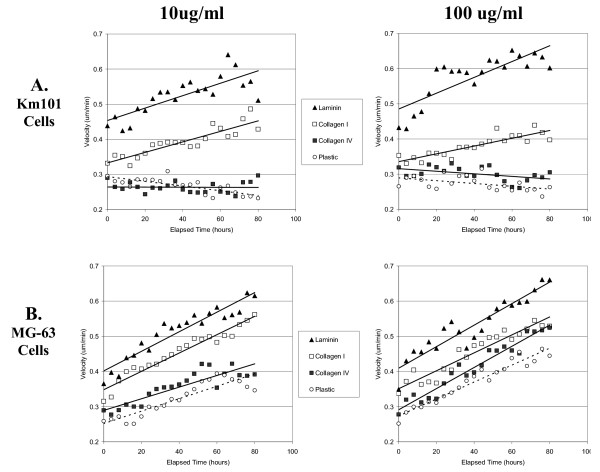
**Kinetics of velocity response**. Velocity measurements were averaged for KM101 cells (A) and MG-63 cells (B) at coating concentrations of 10 ug/ml (Left Panels) and 100 ug/ml (Right Panels). Each point represents the average for 8 images (acquired every 30 minutes over four-hour time intervals) from triplicate wells in each of 3 experiments. Surface coatings are indicated as follows: laminin (triangles), collagen I (open squares), collagen IV (closed squares), and mock-coated plastic (open circles). Linear trend lines were added, and statistical analysis was applied as described (Methods). For KM101 cells at dose level 10 ug/ml (upper left panel), the estimated velocity model is: Collagen I: v = 0.3327+0.0015*time; Collagen IV: v = 0.2641-0.00002*time; Laminin: v = 0.4532+0.0018*time; Plastic: v = 0.2938-0.0007*time. The p-values for the intercepts and slopes are all significantly positive (p < 0.0001) except that the slope for Collagen4 is nonsignificant (p = 0.9217). The overall test for the equivalence of the starting velocity is significant (p < 0.0001), which means not all the starting velocities are equal, and the Bonferroni adjusted pair wise tests show that all the initial velocities are significantly different. The overall test for the equivalence of the slope is significant (p < 0.0001), which means that not all the slopes are equal, and the Bonferroni adjusted pair wise tests show that only Collagen I and Laminin have equal slope (p = 0.5100). For KM101 cells at coating concentrations of 100 ug/ml (upper right panel), the p-values for the intercepts and slopes are all significant. The overall test for the equivalence of the initial velocity is significant (p < 0.0001), which means not all the initial velocities are equal, and the Bonferroni adjusted pairwise tests show that only Collagen I and Collagen IV have the same initial velocity (p = 0.0202). The overall test for the equivalence of the slope is significant (p < 0.0001), which means not all the slopes are equal, and the Bonferroni adjusted pairwise tests show that only Collagen IV and Plastic have the same slope (p = 0.8705). For MG63 cells at dose level 10 ug/ml (lower left panel), the p-values for the intercepts and slopes are all significant (all p < 0.0001). The overall test for the equivalence of the initial velocity is significant (p < 0.0001), which means not all the initial velocities are equal, and all the Bonferroni adjusted pair wise tests show significant difference. The overall test for the equivalence of the slope is significant (p < 0.0001), which means not all the slopes are equal. The Bonferroni adjusted pair wise tests show that Collagen I and Laminin have the same slopes (p = 0.5176), Collagen IV and Plastic have the same slope (p = 0.6623), and other pairs have significantly different slopes. For MG63 cells at 100 ug/ml (lower right panel), the p-values for the intercepts and slopes are all significantly positive (p < 0.0001). The overall test for the equivalence of the starting velocity is significant (p < 0.0001), which means not all the starting velocities are equal, and the Bonferroni adjusted pair wise tests show that only Collagen IV and Plastic have the same initial velocity (p = 0.0428).). The overall test for the equivalence of the slope is significant (p = 0.0084), which means that not all the slopes are equal, and the Bonferroni adjusted pair wise tests show that only Collagen IV and Plastic have significantly different slope (p = 0.0042), others are not significant.

Automated quantitation demonstrated that cells responded to the higher concentration laminin coatings with increased cell velocity over an elapsed time period up to three days, at which time in one of three experiments cells exceeded the 30% confluence cut-off for velocity measurement (Figure 4). A significant but less pronounced effect for collagen type I coating in comparison to mock-treated plastic and collagen type IV coating was also evident. Both cell types exhibited higher initial velocities on laminin and collagen type I surface coatings compared with plastic, even during the initial 4-hour time interval.

A conspicuous feature of these results is that MG-63 cells accelerated throughout the time period regardless of surface treatment and even on mock treated plastic, whereas KM101 cells exhibited constant or slowly declining velocities on mock coated plastic and on collagen type IV coated surfaces. The continued acceleration of MG-63 cells may be verified visually in time-lapse images [see Additional file 2], but this behaviour was not perceptible to the unprepared eye prior to quantitative analysis. The kinetic data output from the automated system provides an obvious advantage in this regard for screening and discovery purposes over non-kinetic methods such as the transwell assay. Statistically significant effects were observed for collagen IV in comparison to plastic based upon the entire data set from three experiments over 80 hours elapsed time (see Figure 4 legend), even though measurement values frequently overlapped between these two groups. Once again, sufficient data are provided by the automated system to demonstrate moderate effects that are not likely to be significant using other methods.

### Velocity dose-response

**Figure 5:**
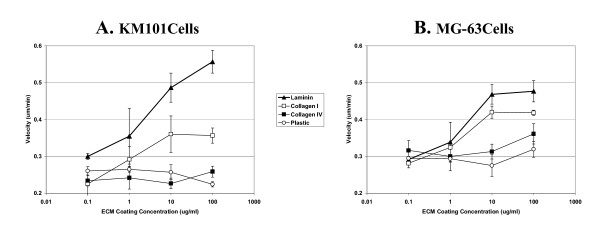
**Velocity dose-response**. Velocity measurements for single cells were averaged over the time period from 24 to 48 hours and were normalized based upon the average velocity of cells on mock-coated plastic in each of three experiments (see Methods). Velocities are plotted versus the coating concentration of the extracellular matrices laminin (triangles), collagen I (open squares), collagen IV (closed squares), and mock-coated plastic (open circles) for KM101 cells (A) and MG-63 cells (B). Error bars indicate the standard error for three experiments. Identical mock-coated plastic controls were included for each concentration; in this case, since the concentration values for plastic are bogus, these results illustrate random variation for the replicate means.

Dose-response curves indicate that KM101 cell velocity increased approximately 0.1 um/min with each 10 fold increase in laminin coating solution concentration across the range of concentrations tested, whereas for collagen type I, the cell velocity appeared to maximize at approximately 0.1 um/min above control levels with the 10 ug/ml coating solution (Figure 5). For MG-63 cells, more modest increases in velocity were observed. Average velocity appeared to maximize at levels approximately 0.15 um/min and 0.07 um/min above control levels with the 10 ug/ml coating solution concentrations for laminin and collagen type I, respectively. Average velocity levels were not significantly increased with collagen type IV coating solutions above those observed for mock-coated plastic for either cell type.

### Velocity measurement heterogeneity

**Figure 6:**
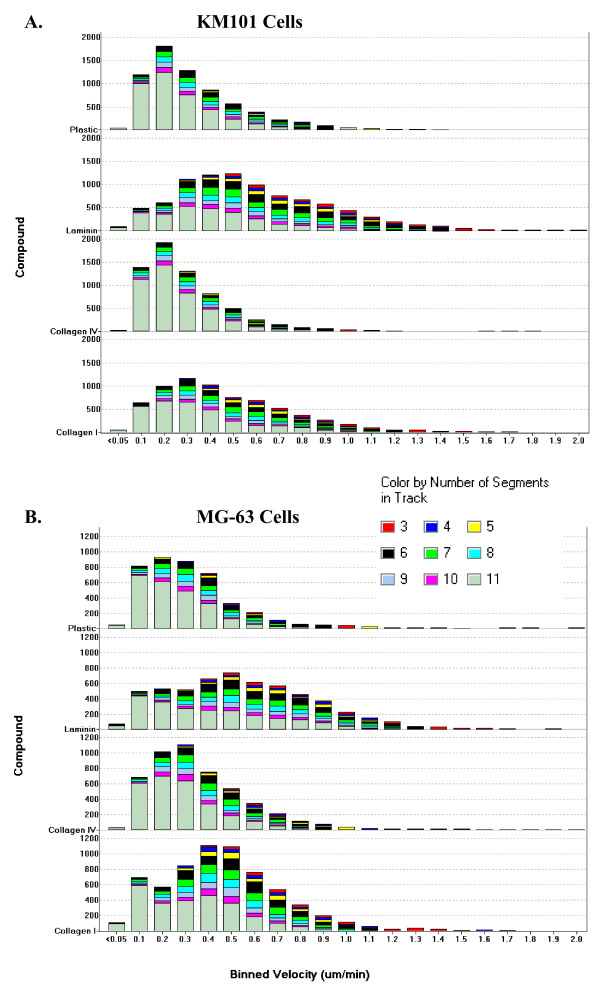
**Velocity distributions**. Histograms show changes in the velocity distribution profiles in response to surface coatings for the different extracellular matrices laminin, collagen IV and collagen I (all at 10 ug/ml coating concentration) in comparison to mock-coated plastic for KM101 cells (A) and MG-63 cells (B). Velocity measurements on individual objects were filtered according to criteria for single cells as described (see Methods) and were compiled over an elapsed time from 24 to 48 hours from three experiments. The horizontal axes indicate velocity measurement bins from which the number of objects included within each bin are determined as shown on the vertical axis (faint horizontal lines represent 1000 objects). The different colours within each bar indicate cell track length (from bottom to top: grey indicates tracks of 11 or more segments, violet: 10 segments, light-blue: 9 segments, bright-blue: 8, green: 7, black: 6, yellow: 5, dark-blue: 4, and red: 3 segments).

There are many possible ways to visualize and to quantitatively summarize the data. For the kinetic and dose-response analyses above, a velocity measurement was included for each object at every time point when the set of criteria were fulfilled, i.e. the object area was in the specified range for each cell type, the track connected across at least 4 points, the track fence size was greater than 20 microns, and the total area occupied by cells was less than 30% of the total image area. The population heterogeneity of these measurements under different treatments over a 24-hour time period is illustrated in Figure 6. The distributions are positively skewed and appear unimodal for KM101 cells but exhibit evidence for bimodality for MG-63 cells, suggesting the possible continued presence of a subpopulation of stationary non-cell objects in the measurements. Under the higher area-filtering limit for MG-63 cell objects, larger fences would be expected for stationary non-cell objects; but attempts to increase the fence-size filter resulted in significant loss of records from cell-like objects without eliminating the bimodality (not shown). Velocity measurements up to 2.1 um/min were recorded. Each measurement represents an average over at least three segments (1.5 hours) up to as long as 10 segments (5 hours); the relative contribution from tracks of each size for each bar in the histograms is indicated by colours (see Figure 6 legend). It is evident that a significant proportion of tracks were longer than 10 segments and that shorter tracks were more numerous in the velocity range associated with live-cells (≥ 0.2 um/min) as opposed to the range associated with non-cell stationary objects (0.1 um/min; see Segmentation/Outline Error below). At similar cell densities, more rapidly migrating cells are more likely to terminate tracks by colliding or leaving the viewfield [see Additional file 3].

The individual cell velocity distribution modes were noticeably shifted toward higher velocities with laminin and to a lesser extent with collagen type I surface coatings, in comparison with mock-coated plastic and collagen type IV. These histograms provide a more descriptive picture of the nature of the response of the cell populations to the surface treatments than that provided by measures of central tendency alone, without invoking complex statistical models.

**Figure 7:**
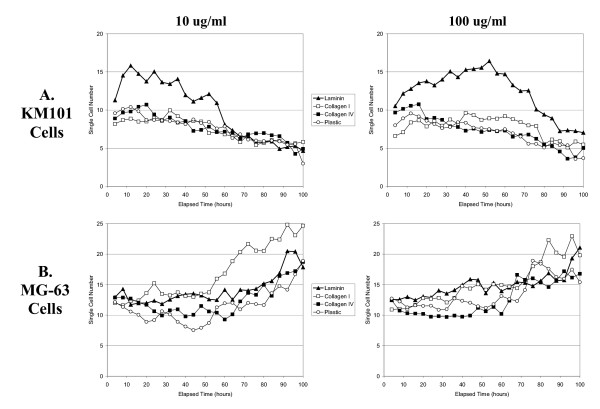
**Single cell numbers**. The average number of objects that met the criteria for measurement of velocity (see Methods) over 4-hour time intervals for KM101 cells (A) and MG-63 cells (B) at extra-cellular matrix coating concentrations of 10 ug/ml (Left Panels) and 100 ug/ml (Right Panels). Each point represents the average for 8 images from triplicate wells in each of 3 experiments. These numbers represent the typical sample size, n, from the populations of measurements shown in Figure 6, from which a mean velocity is calculated for each well at each time point. The observed dispersion of these mean velocities is shown in Figures 9 and 10.

To provide sufficient numbers of objects for reliable averaging, triplicate wells were coated and seeded for each treatment/dose/cell type combination. Single cell counts, i.e. the average number of objects identified in each image that met the area-range criteria for single cells, and for which velocity measurements were included in the kinetic, dose-response, and velocity distribution histograms, are shown in Figure 7. There was a tendency for more motile cells to break free of clusters, i.e. to "scatter" [see Additional file 1], and this tendency gave rise to and is reflected in the greater number of single cells on laminin coated surfaces. For KM101 cells, single cell numbers eventually declined as crowding from cell growth contributed to a greater proportion of cells in clusters. For MG-63 cells, slightly increasing numbers of single cells were observed despite growth toward increasing levels of confluence; this increase correlates with, and possibly resulted from, accelerating motility [see Figure 4 and Additional file 2].

**Figure 8:**
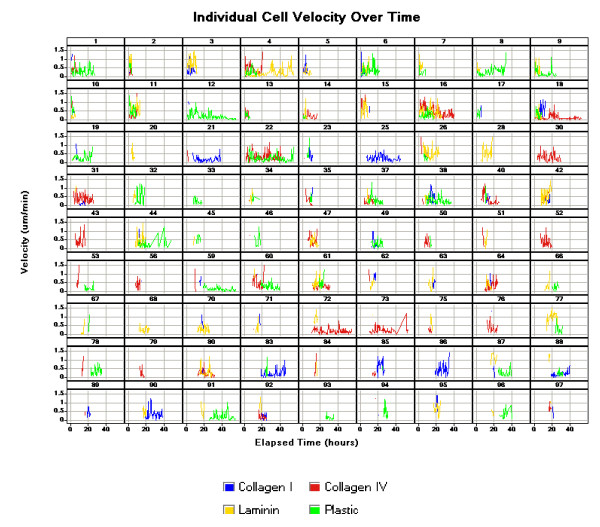
**Individual cell velocities over time**. Instantaneous velocity for individual KM101 cells is plotted for each time point (30 minute intervals) across time periods that extend in each case from the initiation to the termination of individual cell tracks. Cell tracks from four wells with different surface coatings (Collagen I – blue, Collagen IV – red, Laminin – yellow, Plastic – green) are shown; coating concentrations were 100 ug/ml in each case. The number at the top of each frame indicates the "unique track index" by which the tracks are identified. Not all surface coatings are represented in each frame, because in many cases tracks that were initiated did not meet criteria for track length, fence size, or object area (see Methods). These plots show the magnitude of individual non-smoothed velocity vectors, indicating cell motion plus random contribution from stage noise and cell-outline error at each 30-minute time point. In many cases the same cell may terminate in one frame and reinitiate in another due to collision.

Alternative approaches to data analysis can be used to examine the heterogeneity of velocity measurements at the individual cell level over time, and to ask such questions as: Do subsets of fast and slow cells exist, or do all cells show a wide range of speeds over time? How long can a rapidly moving cell maintain rapid motion? Is there evidence that individual cells require resting periods? How does cell-cycle phase affect velocity? An example of velocity histories at the individual cell level is shown in Figure 8. More specific analysis at this level may form the basis of a subsequent report; findings of the current study afford an ability to assign significance levels to the instantaneous peaks and troughs of such non-smoothed data (see below).

### Technical precision and quality control

**Figure 9:**
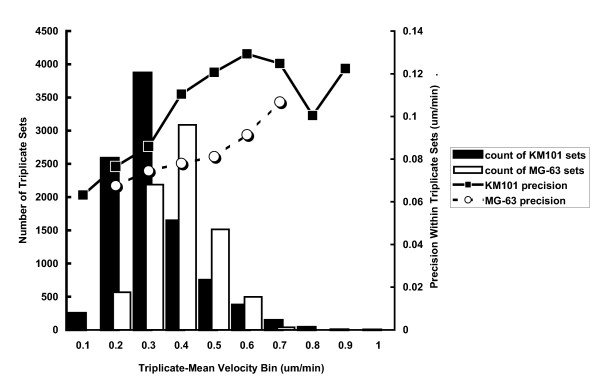
**Precision between replicates**. Precision (right-hand y axis scale) of the mean velocity from all cells in an image at a single time point is represented by the square root of the average variance from triplicates ranked according to mean velocity for either KM101 cells (square symbols – solid line) or MG-63 cells (round symbols – dashed line)(See Methods section). The bars in the background represent the number of triplicate sets that were included in each velocity bin (x axis) for determining average variance for KM101 cells (black bars) and MG-63 cells (white bars). The reliability of the precision estimates is reinforced by the smooth correlation of precision estimate with mean velocity across much of the range. Note how precision estimates became more "unstable" as the number of triplicate sets (bars in background) decreased at higher mean velocities.

The performance capabilities of the automated system, including inter-well variation that is associated with plate manufacture, pipeting, and other operations, may be examined in terms of the technical precision of measurements between replicate wells within experiments (Figure 9). The abundance of data that can be automatically gathered without constraint over time (in contrast to manual analysis by graduate students), leads to robust precision estimates, shown here to be proportional to velocity measurement values.

**Figure 10:**
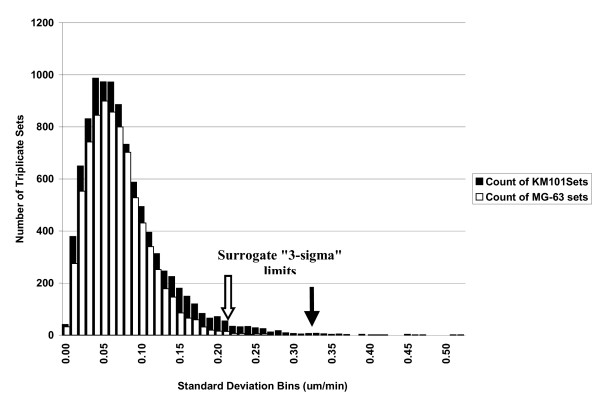
**Graphical quality control limits**. The full population of triplicate sets shown in Figure 9 is here represented as a histogram of the individual standard deviations for velocities from triplicate wells of KM101 cells (black bars) and MG-63 cells (white bars). Presented in this fashion, the data are useful for graphically estimating quality control limits and identifying outliers. Data from images of MG-63 cells are shown to be "well behaved" in comparison to KM101 cells, which exhibit a number of outliers to the right of the "normal" limit expected for the tail. Arrows indicate positions of "3 sigma" limits that are based on the observed percentage (99.7%) of observations (rather than on a more complex transformation of the data according to a mathematical model). In the case of MG-63 cells, the limit (block arrow) excludes several adjacent triplicate sets that are properly part of the distribution. Two certified MG-63 outliers are not readily visible on this chart at 0.40–0.41 um/min. In the case of KM101 cells, the "3-sigma" limit (solid arrow) more haphazardly cuts off a chain of outliers. As described in the text, one source of KM101 outliers was identified by manual viewing of tracked objects in the image sequences, and may lead to a rational filtering method that would improve the data on both sides of the control limit for KM101 cells. Since the average variance for triplicate velocity measurements correlated to some extent with average velocity (see Figure 9), a more refined approach to quality control would involve first ranking the data according to average velocity. Presumably the next step would be to determine whether a single member of the outlying triplicate sets should be discarded in favour of preserving the remaining two measurements in each case.

Since the velocity data are not normally distributed (bounded on the left and skewed toward the right), we examined histograms for standard deviation from triplicate sets as an approach to graphical estimation of quality control limits (Figure 10). For MG-63 cells, all replicate data appeared to be "well contained" by an upper bound of 0.26 um/min, with only two outliers (at ≥ 0.4 um/min) among almost 8,000 triplicate sets. For KM101 cells, 99.7% of the data were contained within an upper limit of 0.32 um/min, leaving 33 questionable sets out of almost 10,000 triplicate sets. Manual examination of some of these sets revealed the presence of surface blemishes in close proximity to each other that gave rise to oscillating tracks between them in images from one well of the set. The regularity in these false tracks may provide a mechanism for filtering them out of the larger data set in future experiments. These data establish a benchmark for monitoring quality in future studies and for inter-experiment and inter-lab comparisons.

### Segmentation/outline error

**Figure 11:**
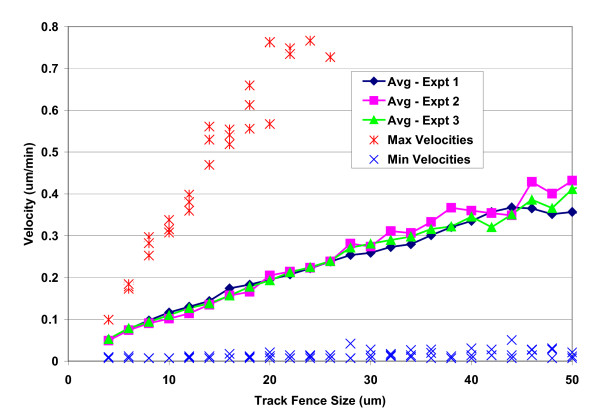
**Velocity at small fence sizes**. Average velocity (lines with symbols), individual maximum velocity (*), and minimum velocity (×) measurements are shown for objects associated with tracks of small fence sizes from three experiments (see legend). Average velocity was calculated as the mean of all velocity measurements from objects meeting cell specific criteria for area with tracks consisting of 3 or more segments, pooled according to fence size and experiment. Consistency between experiments reflects consistency in the rate at which stage noise and object segmentation variability are exhibited. By limiting fence size, we also limit the maximum possible displacement, and we would expect this to be reflected in the maximum observable velocity. It is. Consider tracks with a fence size of 20 microns (10 pixels); this is near the maximum observable displacement for these objects between any two time-points*. Since the images were taken at 30 minute intervals, the corresponding maximum permissible velocity is 20/30 um/min, or 0.67 um/min, which corresponds to data from all three experiments at a fence size of 20 um. These data, in addition to manually constructed images (see Methods), confirm soundness within the system for accurate velocity measurement. (*Fence size is not strictly the maximum observable displacement. A slightly more sophisticated definition of fence size would use the diagonal from maximum x and y values, which would result in slightly greater fence sizes for objects displaced in random directions.)

A surrogate for the empirical evaluation of the total variability associated with both stage noise (i.e. imprecision in returning to the same view-field between acquiring sequential images) and object segmentation variability (i.e. imprecision in "drawing" cell outlines) would ideally consist of inert cell-like objects that remained stationary on the surface. In Figure 3 above, we showed that tracks associated with fence sizes below 20 microns arose predominantly from stationary non-cell objects of similar area to single cells, and here we consider using these objects, excluded from the live cell analysis, as logical surrogates for estimating system variability. The average velocities of such objects at each fence size were nearly identical for all three experiments, demonstrating system stability with respect to stage noise and image segmentation parameters (Figure 11).

**Figure 12:**
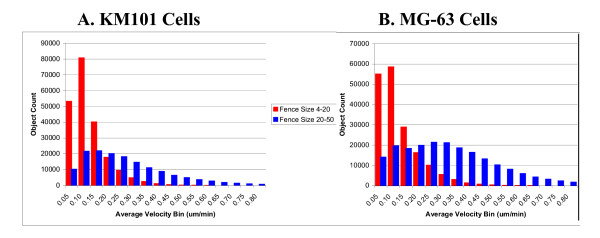
**Velocity histograms at small fence sizes**. The same data that gave rise to the averages and ranges in Figure 11 are represented in histograms here after having been grouped according to whether each velocity measurement was associated with a track of fence size smaller than 20 microns (red bars) or fence size from 20 to 50 microns (blue bars). Measurements were also grouped according to whether the well contained KM101 cells (A) or MG-63 cells (B). A total of over 600,000 measurements arising from three experiments are included. These data should be interpreted in conjunction with evidence presented in Figure 3 for the proportions of non-cell and live-cell objects associated with the group distributions shown here. Note that the MG-63 distribution for the larger fence size population is bimodal, further supporting the dichotomy of object type.

A clearer picture of the proportional contribution to the average velocities from non-cell and live-cell objects is shown by the velocity distributions for fence sizes below 20 microns versus those above 20 microns, respectively (Figure 12). The system velocity output at the individual object level for stationary non-cell objects is seen to cluster about a mode of 0.1 um/min, and approximately 90% of the measurements fall between 0 and 0.23 um/min. Taken in conjunction with Figure 4, these data support an estimate of approximately ± 0.1 um/min for the uncertainty in velocity measurements due to stage noise in combination with object segmentation variability.

### Comparison of automated tracking versus manual tracking

**Figure 13:**
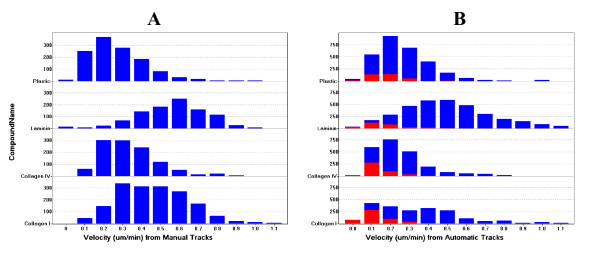
**Velocity histograms by manual and automated tracking**. Velocity measurements were acquired through manual tracking (Panel A) or through automated tracking (Panel B) from image sets from four wells as described in the text. Different coating compounds are indicated along the y-axis (all 10 ug/ml). Red portions of the bars for automated tracking indicate measurements that came from tracks with fence sizes of less than 11 pixels (i.e. 20 um or less motion in the x or y direction over the length of the track). These tracks (red portions) were selected for exclusion based upon the supposition, supported by Figure 3, that such tracks arose predominantly from non-cell stationary objects. Note in particular how subtraction of the red portions from the 0.0, 0.1, and 0.2 um/min bins for Collagen IV and Collagen I from the automated track histogram (panels B) would bring the remaining data (blue portions) more in line with the profile of data from manual tracks (Panel A).

Comparison of the results from automated tracking with manual tracking further validated the accuracy of the system, the efficacy of the fence size filtering method, and the individual fidelity of cell tracks from example image sets. Velocity histograms prepared from all data obtained by both methods show that similar treatment effects were obtained (Figure 13). Moreover, if we assume that the velocity distributions acquired from manual tracking are more truly "accurate" because only known live-cells were tracked, then this comparison further supports the validity of the "fence-size filter" method to discriminate non-cell stationary objects. This is because removal of the red, small-fence-size data from the histogram bars for automated tracks (Panel B) brings them more in line with the manual track histograms (Panel A). Thus, this comparison shows that we are not excluding appreciable numbers of stationary live cells by applying the fence-size filter, and that the automated data for velocity is very similar in quality to that obtained manually.

**Figure 14:**
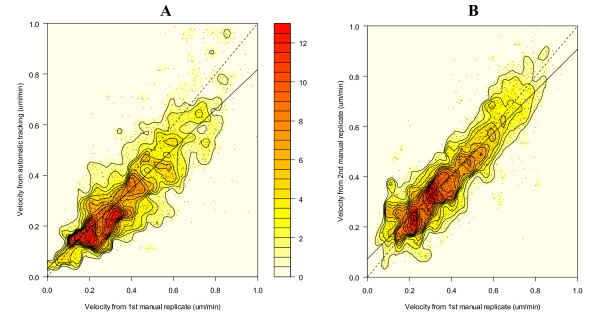
**Correlation between manual and automated velocity measurement**. Velocity from the automated method (Panel A) or from the second manual replicate trial (Panel B) were plotted in reference to corresponding matched velocities from the first manual tracking exercise (y axis = dependent variable, x axis = independent variable). Increasing frequency of points is indicated by density contour lines and red shaded intensity (color scale). The dashed line indicates the line of identity and the solid line indicates the regression line for each comparison. Regression statistics were as follows: For automated tracking (y) versus the first manual tracking (x) (Panel A), the slope and intercept were 0.79 ± 0.02 and 0.03 ± 0.01, respectively with Pearson's correlation, r, equal to 0.81 ± 0.01. For the second (y) versus the first (x) manual tracking (Panel B), the slope and intercept were 0.84 ± 0.02 and 0.07 ± 0.01, respectively with Pearson's correlation equal to 0.86 ± 0.01.

To more specifically compare manual versus automated tracking on a point-by-point basis, we developed an algorithm to match object positions from automated analysis with manually tracked cell locations from each image. A necessary component of this comparison is determining the precision of each method, and so both methods were performed in replicate on the identical image sets. Standard statistical methods of comparison were applied to the matched velocity data. Regression analysis for a perfectly precise system would yield slope equal to one and intercept equal to zero, producing a 45° line of identity in an x-y plot. Such a result was obtained for replicate analyses by automated tracking, i.e. almost 20,000 individual cell locations were identically analysed when the original "jpeg" images were reprocessed. When comparing the test system versus a reference system, an observed slope *less than *one and intercept *greater than *one for a regression line would imply that the dependent variable is "accurate" in a mid-region, but is somewhat overestimated at low values and underestimated at high values. These features were evident from regression analysis of automated versus manual tracking, but also for the second replicate versus the first replicate manual tracking exercise, pointing out limitations in the precision of manual tracking as a reference method (Figure 14).

To better understand the magnitude of these errors in practice, we examined the actual differences in locating cell positions by the automated and manual tracking methods. Our data set consisted of 716 cell positions that were co-located by automated tracking and by the first and second manual exercises according to the matching algorithm (see Methods). No single position can be considered the "true" position; however, the differences between replicates can be used to estimate precision, S, according to the relationship, S = 1/v2 times the standard deviation of the differences, where the differences retain the sign; for example, if the second comparison measurement is larger than the first, the sign is negative. By this approach, for the first and second manual exercises, the precision was 1.91 and 2.03 pixels in the x and y coordinates, respectively. For the comparison between automated tracking and manual tracking the precision ranged between 2.04 to 2.28, but when automated tracking was compared with the mean from the two manual tracking exercises, the precision improved to 1.87 and 1.91 pixels in the x and y coordinates, respectively. Thus, the automatically determined cell positions were closer to the mean positions from the two manual determinations than any individually determined position was to any other. Under the settings used, a distance of 2 pixels corresponded to an error of less than 1/32 inch, easily within the realm of human error for mouse-clicking on a monitor.

**Figure 15:**
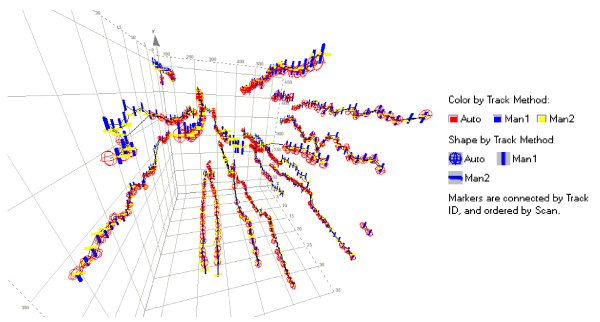
**3D visualization of matched cell tracks**. Cell positions from the first manual tracking exercise (vertical blue markers) that matched either the second manual tracking exercise (horizontal yellow markers) or the automated tracking (red globes) were plotted in 3D with x and y coordinates in the plane of the paper and with increasing elapsed time (scan = 30 minutes) represented by the z axis coming "up" toward the viewer (similar to Figure 2). Lines connect markers by unique Track ID numbers, so that detailed features of track continuity may be examined. For clarity, only cell positions from one example culture well are shown (mock-coated plastic).

Finally, the time-resolved fidelity of individual tracks may be examined in detail in 3D scatter plots of the x and y locations over time (Figure 15). Here we show all cell positions for which the first manual tracking matched automated tracking and/or the second manual tracking exercise. Replicate manually tracked cell positions are represented along the full length of most tracks. Automatically tracked cell positions show more frequent gaps resulting from merging and branching, but they are nevertheless represented along the majority of the positions for most tracks. Altogether, these data provide ample support for the accuracy and fidelity of the automated tracking method.

### Cell proliferation

**Figure 16:**
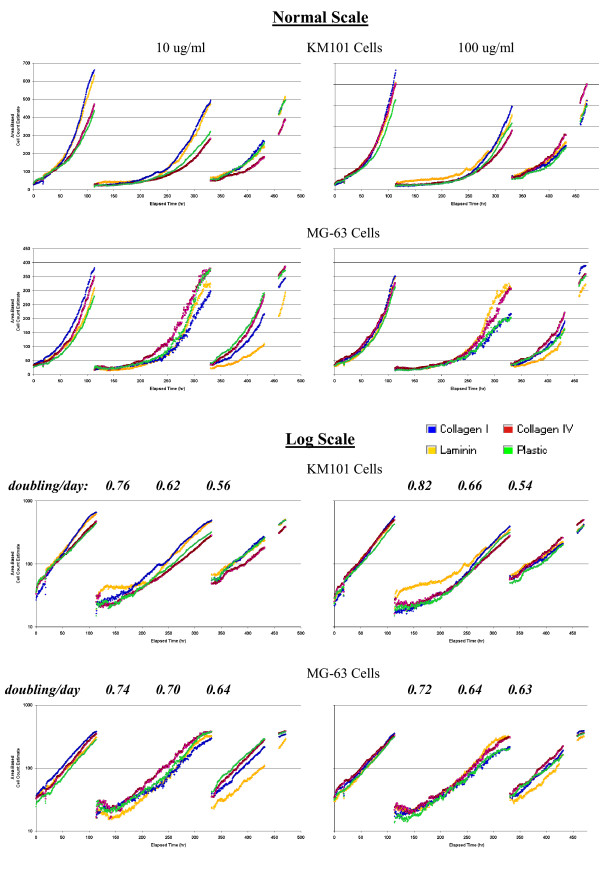
**Growth curves**. Area-based cell numbers (see Methods) were plotted versus time (hours) over the course of the three experiments performed for this study. Data from wells with extra-cellular matrix compound coating concentrations of 10 ug/ml (left panels) and 100 ug/ml (right panels) are shown for both cell types, KM101 cells (first and third rows) and MG-63 cells (second and fourth rows) using a normal scale (top two rows) and log scale (bottom two rows) for area-based cell numbers along the vertical axis. A datum point is plotted for each image, i.e. at every 30 minute interval. Each point represents the average from a set of triplicate wells. During the third experiment (far right hand set of curves in each chart), disengagement of the coupling for the mechanical focus was discovered and repaired; the data from that period of malfunction were removed, as evident in the 27 hour gap before the end of the third experiment in each chart. Area-doubling rates obtained from the average of the four treatments for each of the three experiments are printed above the log-scale charts. These area-doubling rates (doublings/day) are the slopes obtained from linear regression of log base 2 cell numbers versus time across time periods selected from each experiment during which the log scale curves were linear – at least 48 hours in each case. Area-doubling rates for the first experiment were significantly greater than the doubling rates for the second and third experiments (t-Test, p < 0.01).

**Figure 17:**
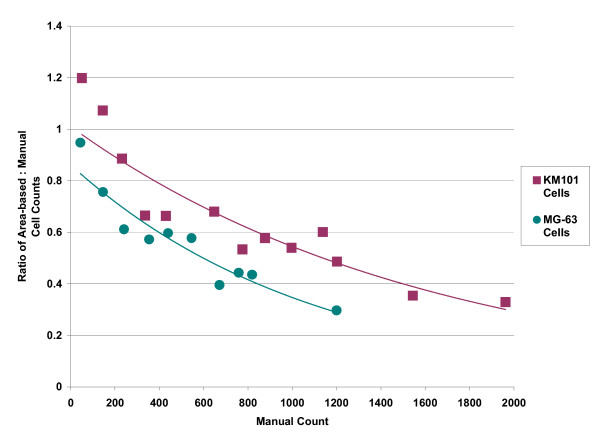
**Ratio of area-based cell counts to manual cell counts**. Area-based cell counts were automatically estimated by dividing the total area of segmented objects in each image by the average area of a single cell for each cell type, KM101 (red squares) and MG-63 cells (green circles). Manual counts were scored using a custom program that marks cells when clicked on, and increments a total count. A total of 90 images were analysed for each cell type across a range of confluence levels. The individual counts were grouped and averaged according to manual count levels; maximum values corresponded to 100% confluence for each cell type. Trend lines were based upon an exponential fit.

As cell numbers increased over time, the total area of the segmented objects naturally increased, and initially we used this sum divided by the average area of separate individual cells for estimation of total cell numbers per view-field. Such numbers are seen to increase exponentially, and the slope of log-transformed cell number can be used to estimate doubling times for comparison of growth rates (Figure 16). The linearity of the log scale plots and the high density of data points permit estimation of area-doubling times, over time periods as short as one to two days during the "clean" exponential growth phase, which persisted for 4 to 6 days. However, quantitative analysis of cell growth rates by the area-based method of estimating cell numbers was set-aside when we compared manually scored cell numbers with area-based estimates (Figure 17). The area-based method increasingly underestimated actual cell numbers with increasing levels of cell confluence.

Regardless of the non-linearity of area-based cell numbers, the monitoring of cell growth based upon total cell area provided extremely valuable information. Within experiments, the log-based slopes were similar for the different treatments, with the pronounced exception of laminin and KM101 cells in the second experiment, even when starting with different initial seeding densities and when absolute cell numbers differ considerably over time as was evident in the three experiments performed for this study. A faster doubling rate for all treatments in the first experiment contrasted with slower doubling in the second and third experiments for both cell types. Treatment-related patterns occurred within experiments that were not evident between experiments, and even without quantitative analysis it is clear that no reliable pattern of surface-treatment effect on cell growth can be found. Hazards are evident, however, if conclusions were to be drawn from a more limited number of experiments, even with convincing within-experiment data.

One can speculate on possible causes for the between and within-experiment patterns shown here. For the second experiment, seeding densities were unusually low, and an atypical initial period of cell death was evident in the time-lapse videos during the first day after plating (data not shown) and is shown also by the unusual "lag phase" in the log-based charts for both KM101 and MG-63 cells in the second experiment. Typically, healthy cells under normal conditions begin exponential growth immediately upon re-plating, as evident in the linear log-based graphs for the first and third experiments from the point of initiation up to the point of confluence. The departure evident for the second experiment in the growth charts holds implications for variability between experiments in the velocity data presented above (Figure 5). Standardization of procedures and testing for robustness are called for. The value of our system for assisting in such efforts is prominently evident in these charts, apart from the question of treatment-related effects on growth in this study. In future studies, consistency in growth parameters could be made a prerequisite for acceptability of results for other parameters; here the results serve to demonstrate system potential.

## Discussion

This study demonstrates the applicability of a fully automated image acquisition and analysis system for quantitative measurement of cell motility and for monitoring cell proliferation in a relatively high-throughput manner. Simultaneous comparison of multiple treatment variables in a 364 well plate format over extended periods of time is possible. Here we show how various surface coatings of extracellular matrix compounds can induce increased velocity in two types of osteogenic cell lines, how these responses vary over time periods of more than three days, and how they vary by surface coating concentration, i.e. dose-response. We show that laminin and to a lesser extent collagen type I surface treatments brought about increased migratory activity, while collagen type IV did not, in comparison to mock-coated plastic for KM101 cells and MG-63 cells. We show further how the extensive multiparametric output can be used to selectively isolate data of interest for improvement of bio-informational content and for characterization of system performance.

We believe our system is the first to accomplish full automation of time-lapse motion analysis of cells in culture, broadening the scope of application well beyond the practical limits imposed by manually interactive methods. Image sets consisting of hundreds to thousands of scans from up to 384 locations over extensive time periods (days to weeks) can be acquired and batch processed, first to segment cell-like objects and clusters of such objects, and subsequently to construct tracks for the segmented objects over time. Quantitative data is seamlessly exported to a Sequel Server Data Base from which results are presented in various forms as shown here. The relational data base is absolutely necessary for handling the large volume of multi-parametric data – over three million records for this study, including one for each object at each time point containing more than 40 fields of positional, textural, morphological, and motility-related data. Additional fluorescence measurements are also commonly used [19-21].

Our analysis contrasts in some respects with the exquisite optical characteristics and advanced analytical details of migratory activity achieved in systems where parallel plane surfaces are provided by specially constructed chambers [e.g. [25,26]]. Such non-conventional culture environments overcome the meniscus problem, but are not readily adaptable to simultaneous analysis of multiple treatment conditions in a high throughput manner. Most importantly for screening and discovery purposes, our system preserves the essential features of trajectory analysis that were emphasized by Friedl and co-workers for evaluating subtle changes that reveal treatment effects on cell locomotion [27,28].

To support the introduction of fully automated cell tracking, we have included a thorough analysis of measurement variability, system errors, and accuracy. Biological differences between individual cells dominated the velocity measurement heterogeneity for the cells chosen for this study, while stage noise, cell segmentation, and cell positioning were shown to be relatively minor sources of error. For more slowly moving cells, i.e. those with velocities less than 0.4 um/min, the latter relatively minor errors will result in large coefficients of variation under current conditions. Rational data-filtering methods, such as exclusion of data from non-cell stationary objects based upon "fence size" and exclusion of data from objects with areas outside of single-cell ranges (see Methods), yielded further improvements to the detectability of biological response from single cells. The accuracy and validity of these approaches were supported by comparison of the automated data with data from manually tracked cells. A significant feature of this analysis was the finding that, in general, automatically determined cell positions were closer to the mean positions from manual tracking than the replicate manual positions were to each other. Thus a major limitation of this comparison was the imprecision of manual tracking.

On the other hand, the perfect precision of automated tracking represents a two-sided coin. It will precisely repeat any errors, posing an obstacle to data control based upon monitoring internal consistency. At the same time, small variations in processing variables can be readily applied, tested, and evaluated. For example, in current experiments, we are exploring the possibility of obtaining better "average" cell positions by acquiring replicate non-identical images closely spaced in time, e.g. one minute apart, for characterizing motion over longer intervals, typically 30-minutes. Such an approach should improve precision and segmentation reliability at the individual cell level, while simultaneously providing a foundation for routine control based on inherent data consistency. The ability to perform automated batch processing will in due course be utilized more broadly for quality control and for optimization of image processing variables. Advantage can be leveraged from perfect precision by applying and evaluating systematic changes to variables of a contrast enhancement algorithm, for example, followed by repeated cell segmentation and cell track processing of representative image sets, such that cell track lengths, in terms of linked segments, would be maximized for non-collision-related scenarios. Automated routines for such optimization are not yet implemented, but are entirely feasible.

In seeking higher levels of automation, however, it is important to continue to manually monitor for and evaluate consistency between the raw data input and the processed data output. For each cell type, and for varying treatment conditions, processing variables are checked and evaluated. With each experiment, we manually verify that cell segmentation and cell track formation proceed reliably throughout representative regions of the image sets. The processing software presents the operator with cell outlines and tracks as shown in Figure 1 [see also Additional file 3], and Spotfire™ visualization software is used extensively to verify consistency in the quantitative data. Tracking patterns dominated by short fragments indicate, for example, problems requiring adjustment in image processing variables. Representative image sets are first examined for such problems before initiating batch processing of the full experiment; but it is not impractical to automatically reprocess image sets from an entire experiment when evidence demonstrates a need for it. It should be pointed out that most of the refinements in data filtering, thresholding, and so on for this study were conducted at the level of data base querying, not at the level of image processing or track construction. Whereas image processing requires hours to complete for a full experiment image set, data base queries can be run and re-run in a matter of minutes, and so the effect of changing thresholds or "gates" may be readily examined and interpreted using statistics or visualization software such as Spotfire™. Part of the beauty of automated analysis lies in our ability to make incremental improvements in both image processing and data querying approaches over time, leading to increasingly more accurate and reliable data. In contrast, manual analysis will remain decidedly imprecise and variable over time.

Quantitative results from other published studies further support the general accuracy of our system. For example, Huttenlocher et al. reported speeds ranging from 0.2 to 1.5 um/min for individual CHO cells plated on fibrinogen coated surfaces and later reported average speeds of approximately 0.3 um/min for myoblasts on fibronectin using manually-assisted computer based methods [9]. Similarly, Ware et al. reported maximal average velocities of a 3T3-derived cell line of approximately 1.5 um/min and presented histograms for individual cell velocities ranging from near-zero to approximately 2.5 um/min on a mixed extracellular matrix derivative in the presence and absence of epidermal growth factor [10]. It has been shown that experimental conditions such as pH and temperature, among others, can have a profound influence on motility [29], nevertheless, reported velocities for adherent cells in other published studies are consistent with our findings.

Determining cell proliferation using area-based measurement was shown to be vulnerable to non-linearity between area and cell numbers. But area-based growth curves exhibit exponential characteristics of high quality, and so automated quantitative monitoring of total cell area can be highly informative and useful. Image processing algorithms for segmentation of individual cells within colonies or clusters using transmitted light will yield more reliable data as improvement continues with auto-focusing and contrast optimisation. The use of nuclear fluorescence staining is a reliable alternative for automated cell counting, but known stains are toxic and therefore were not used in this study. Application of a mathematical adjustment factor based upon 'calibration' with manual or fluorescent counts might be considered; the risk here is that treatment effects could be masked, so this approach has not been used.

Velocity enhancement of osteogenic cells with extracellular matrix compounds provided a useful illustration for this pilot investigation of the automated system. It should be pointed out that collagen extra-cellular matrices consist of a 3D network of polymerised fibers, and that the 2D non-polymerised surface coatings used here may not reveal representative cellular response to the normal polymerised form of the extracellular matrix. An unexpected finding from this study was the continued acceleration of MG-63 cells on mock-coated plastic as well as on the extracellular matrix compound coated surfaces in contrast to KM101 cells that did not accelerate on mock-coated plastic or collagen type IV coated surfaces. Trypsinization cleaves cell adhesion receptors at the time of replating, and it is possible that the re-synthesis and transport of such receptors played a role in the kinetics of the responses shown in this study. The effects of such recovery would not seem to explain the continued acceleration of MG-63 cells through several cell cycles, however. Migration inducing-factors secreted by osteoblasts have been reported [6,30], and it is possible that an effect from such a factor is involved here. Alternatively, MG-63 cells may secrete extra-cellular matrix proteins that gradually accumulate on the surface to enhance migration rates.

## Conclusion

This study demonstrates performance of a system for fully automated, time-resolved, and high throughput analysis of cell migration and proliferation, applied to continuously growing cultures in standard 384-well plates. This format permits the study and comparison of cellular responses to a large number of different treatment conditions, as shown here for three different extracellular matrix coatings on cultureware plastic for two different osteoblast-type cell lines. The simultaneous determination of multiple parameters at the individual cell level affords opportunities for data-base filtering and mining in order to select biologically relevant information. The automated velocity analysis has been demonstrated to be accurate within a level of uncertainly that is imposed by manual analysis of cell velocity from time-lapse image sets. Data quality and system performance measures have been developed that can serve as a basis for the design and quality control of large scale experiments for which this system is broadly applicable.

## Methods

### Cells and cell culture

MG-63 cells were obtained from ATCC and were grown according to recommended protocol (ATCC). Specifically, MG-63 cells were cultured in Minimum Essential Medium (Gibco/Invitrogen) with 2 mM L-glutamine and Earle's BSS adjusted to contain 1.5 g/L sodium bicarbonate, 0.1 mM non-essential amino acids, and 1.0 mM sodium pyruvate with 10% heat-inactivated fetal bovine serum (FBS). KM101 cells, a human bone marrow stromal cell line [22] were grown in 10% FBS in Iscove's Modified Dulbecco's Medium (Gibco/Invitrogen). Cells were cultured at 37°C in humidified atmosphere with 5% CO2. Prior to plating, cells were trypsinized with 0.25% trypsin (Gibco) and resuspended at a density of 2000 cell/ml. Aliquots of 60 ul were seeded into 384 well plates (Costar, black wall) yielding approximately 5 to 15 individual cells in the camera viewfield at the outset of imaging.

The plate layout included triplicate wells for each compound at each coating concentration for both cell lines. All operations were performed using multi-tip pipettors so that inter-well variations within-treatments were minimized to the greatest possible extent. Although the ideal plating pattern would be fully random, for practical purposes, advantage was taken of the interspacing of wells that occurs using 96 well multi-tip pipettors for applying solutions from 96 well "seed" plates into every-other well of the 384 well plate, producing "checker-board" patterns that intermixed treatments, doses, and uniform mock-coated control wells across the plate. Cell suspensions were pipetted from solution basins uniformly across each row.

### Extracellular matrix surface coating

Mouse laminin I (Cultrex), mouse collagen type IV (Cultrex) and collagen type I (Sigma) were stored and reconstituted according to the manufacturer's instructions. Dilutions for coating were performed either in sterile water for collagen types I and IV or in Iscove's Modified Dulbecco's Medium (IMDM; Gibco/Invitrogen) without added serum for laminin I. Control wells were mock-coated using IMDM without added serum. Aliquots of 10 ul of solution were added to each well of a 384-well plate (Costar), and the plate was incubated for 1 hour at 37C. The collagen solutions were aspirated, and collagen-coated wells were allowed to air dry for about 20 minutes in a laminar flow hood. The laminin and mock-coated wells were then aspirated and all wells were rinsed with IMDM and aspirated prior to addition of cell suspensions.

### Cell culture imaging cystem

The cell culture imaging system consists of a custom made environmentally controlled biochamber on an electronically controlled x-y motorized stage driven by stepper motor drive systems (Ludl Electronics, Ltd.) mounted upon an inverted microscope (Nikon TE 300) with electronically controlled motorized focus. The stage moves precisely to each well in the multi-well plate or to any number of locations within each well with a positioning repeatability of ± 1.5 um over the longest distance traveled by the stage. The biochamber temperature is controlled via heating cartridges with temperature feedback loops, and the humidity and CO2 content are controlled via commercial sensors to control feedback loops to a water reservoir heater and low pressure CO_2 _solenoid valve, respectively. The glass windows for illumination and microscope imaging are specifically heated through an electrically conductive indium tin oxide (ITO) coating using feedback temperature regulation so that condensation does not occur on these surfaces. A custom instrument control program, written in Visual Basic and C++, integrates control of the microscope stage, focus, optical filters, shutters, camera, fluidics, image storing functions, thermal zones, and subsystems through a specialized serial interface board with eight RS-232 connections.

### Video time-lapse imaging and analysis

Images were acquired at 30 minute intervals with a 10× objective on a Nikon TE300 inverted microscope with a Photometrics SenSys high resolution (7 × 9 mm, 1036 × 1318 pixel chip) CCD camera (Roper Scientific). Image sequences were processed using a custom software program that identifies and records the location and morphological characteristics of cell-like objects (see Cell Segmentation and Outline Determination). The centroids of cell-like objects are then linked through sequential images to construct "tracks" that trace the route of individual cells according to proprietary algorithms [see Cell tracking algorithm]. The information accumulated during the processing is represented in probabilistic form so that the decision-making process does not have to be "black-and-white" (e.g. is the given object a cell or not, or is the given track the right track or not?), but is postponed until the end of the decision-making chain allowing for corrections for "lost" cells.

In each experiment for this study, 96 or more wells were imaged for at least 3 days, yielding more than a million records, each containing multiple measurements acquired for each object at each imaging time-point, i.e. every 30 minutes. These measurements fall into the categories of motility (scalar and vector forms of velocity, linearity, measures of deviation and frequency of oscillation of cell paths, track size and boundary), morphology and texture (area, perimeter, elongation, eccentricity, roughness, intensity and variation of intensity), summary statistics (object counts, cell counts, apoptotic frequency when fluorescence vital staining is applied) and complex parameters such as cell motility persistence [2], proximity analysis (cell-cell interaction, frequency and duration), division detection, growth rate, and viability (not all are applicable).

### Cell segmentation and outline determination

The optical characteristics for inverted light microscopy of 384-well plates present challenges for robust segmentation of live cells. The meniscus obviates phase contrast; brightfield images are low in contrast and require significant processing. So a succession of filters is used to increase the difference (signal to noise ratio) between the background and foreground (cell-like objects), followed by an efficient region-growing operation that segments cell-like objects from the background. Heterogeneity of illumination across the image is reduced using local histogram equalization. Variations in illumination between images (across time) are handled using histogram matching. Background variations are smoothed using anisotropic filtering and adaptive median filtering, preserving cell detail and texture. Finally with brightfield microscopy, the cell boundary produced by the cytoplasmic membrane easily blends into the surrounding background, so a unique set of gradient variation and texture filters is applied to enhance the cell outline. Following filter-based enhancement, a region-growing operation identifies contiguous areas of cell-like or background-like pixels to segment cell-like objects from the background. A still more involved cell boundary determination can be achieved via active contour techniques (snakes) at the operator's discretion.

### Cell tracking algorithm

The time-lapse interval in multi-well experiments is dependent upon practical considerations including the cell-type specific rate of motion, the total number of wells, the rates of stage movement, camera operation, and so on. For very fast cells such as T cells, imaging is performed on subsets of wells on a rotational basis in order to achieve intervals short enough for reliable track construction. At longer intervals, and particularly for view-fields containing many similar cells, cell track linking across the interval becomes increasingly unreliable because cells change shape and direction frequently, and as their paths converge, incorrect links may be assigned probabilities equal to or greater than correct ones.

Tracking is achieved by linking the matched cell-like objects between consecutive images in a probabilistic manner using a succession of increasingly stringent criteria. First, for each cell-like object, a set of candidate matches is chosen from within maximum speed and acceleration limits. Within this set, cell-like objects (blobs) are assigned to tracks based on match probabilities. Blobs are compared using multiple features such as location displacement, size difference, eccentricity changes, grayscale intensity IQR changes and normalized cross-correlation of the respective image portions. Converting distances into probabilities is done using a Gaussian probability density function. Because we assume that the features are independent, we can also assume that the resulting probabilities are independent. Therefore we can combine the different probabilities,



into the matching probability *P*(*match*_*ij *_| *i*, *j*), where *match*_*ij *_is a candidate match between track *i *and blob *j *and *d*_*n *_is the distance for feature n.

Cell objects in one image may compete simultaneously for multiple matches to different cells in the next image. A rule-based algorithm develops tracks based upon the values of the matching probability and tracking scenario. "Tracking scenario" includes the recent history and circumstances such as cell merging and splitting. For example, when two tracked cells that are similar collide, the merged object cannot be assigned to either track so both tracks are terminated. When two merged cells subsequently split apart, two new tracks are initiated because the matching of cells before and after the collision is ambiguous [see Additional file 3]. The cell tracking algorithm is very efficient in comparison to the cell segmentation; both are completed at the rate of 32 images/min, for 658 × 517 images on an Intel P4 2.8 GHz PC with 512MB RAM.

### Cell velocities

Both magnitude and directional velocity information are output from the linked positions of objects in sequential images. In this study, and for general investigation, we use a scalar average across several time points to smooth variation due to many factors. This average velocity represents the actual distance travelled, as determined by the movement of the centroid of the cell, divided by the elapsed time. Track lengths with fewer than 3 segments were not considered, and a maximum of 10 segments were included such that, for tracks longer than 10 segments, the velocity represents a running average. An exception to this method was used for "instantaneous velocity", as shown in Figure 8, where calculation was based upon displacement divided by time for the single track segment between the previous and current image. In order to exclude objects containing multiple cells in clusters or colonies, only velocity measurements for objects with areas less than three standard deviations above the mean area for each cell type were considered. The mean cell area and standard deviation was 1800 +/- 400 and 3100 +/- 900 square microns for KM101 and MG-63 cells, respectively. The lower limit for object area was 1200 square microns in both cases, based upon optimal visual exclusion of non-cell objects during set up of image processing variables. Velocity measurements were not included when the total area occupied by cells was greater than 30% of the viewfield area, i.e. when the cells were greater than 30% confluent. Finally, in order to filter out plate surface imperfections and adherent particles that gave rise to cell-like objects, data were excluded from tracks with fence sizes of less than 20 microns (See Results).

The technical accuracy of the imaging processing and data conversion steps were verified manually by constructing idealized image sequences with objects "seeded" at known pixel distances in order to generate known velocities based upon typical magnification and binning settings. Microscope optical magnification levels have been verified and calibrated with image pixel dimensions using a standard reticle.

### Technical precision and quality control

Technical precision is here defined as the square root of the average variance for sets of triplicate velocity measurements (each measurement representing the mean velocity for all cells in the image) at single time points. Since absolute precision tended to increase in value for wells with cells of higher average velocity, and since there were sufficient data for analysis across the full range of velocities, we calculated summary precision values for triplicate sets ranked according to their measurement means (Figure 9). These data may be interpreted to indicate that the uncertainty in the mean velocity for a single well at a single time point ranged from approximately ± 0.08 um/min at the 0.2 um/min mean velocity level (CV = 40%) to approximately ± 0.12 at the 0.8 um/min level (CV = 15%) for KM101 cells, and it was slightly better for MG-63 cells. Sources of this measurement variability include the irreducible variation expected from random sampling from populations shown in Figure 6, taking into account the number, n, of cells in each sample, i.e. the number of cells imaged in each view-field as indicated in Figure 7.

Incidentally, the bin-based population variance, in which these precision estimates are rooted, should be employed when using the t-Test, rather than the variance of individual sets of replicates, to evaluate the significance of differences observed within experiments between triplicate means, i.e. for testing whether cell velocity was affected by an experimental treatment compared to a control treatment. The reason for this is that the sample mean and sample variance are independent when sampling from a normally distributed population. In other words, counter-intuitively, the mean of a set of three replicates that are widely spaced is likely to be as close to the "true value" as the mean of a set of replicates that are very closely spaced. The abundance of measurements made available by automation supports the validity of this claim, and would allow for slight adjustment of this principle when the data depart from normality, as it does here.

### Stage noise

At the most fundamental level, because the stage mechanism re-centers each well into view after each time interval, images and data derived from them are subject to random errors associated with slight misalignment of the culture plate at each scan. The limits of this misalignment were determined by expanding images and manually tracking the motion of highlighted features of small imperfections on the culture surface throughout example image sequences. Such features were confined within a boundary of 3 square pixels over greater than 100 sequential images in all three experiments. Assuming Gaussian statistics, a limit of less than 1 pixel was estimated for the standard deviation of alignment of a single-pixel object, i.e. "stage noise". Under the magnification (10×) and binning conditions (2 × 2) used in these experiments, one pixel corresponds to 2 microns; this displacement for an object within a 30 minute time interval yields an upper limit for velocity of approximately 0.07 um/min. This calculation represents an upper limit to the contribution of stage noise, because in practice, stage-positioning error is expected to be a relatively small component of the total error that includes image-processing variability in determining cell outlines and object centroids.

### Comparison of manual and automated tracking

Manual tracking was performed with a custom viewing program that enabled the user to store x and y coordinates by clicking on cells in sequential images with a computer-mouse. The 517 × 658 pixel images were displayed at approximately 7 × 8.5 inches on the monitor (approximately 75 pixels/inch) with a zooming option. A total of 27 cells were manually tracked from each of the four selected treatments. (For this exercise, a 27-cell limit was imposed by the nature of data output into a Microsoft Excel worksheet). The "rules" for manually placing the "centroid" and for terminating or initiating tracks were somewhat discretionary, e.g. the author continued to manually track cells through periods of contact with other cells, even though such scenarios were expected to involve track termination and re-initiation by the automated tracking algorithm [see Additional file 3].

For comparison of tracks on an object-by-object, point-by-point basis, we developed an algorithm to match objects from automated image analysis with manually tracked cells from each image. This algorithm identified and tabulated data from objects with automatically located centroids that fell within 10 pixels (20 um) of the x and y coordinates of the manually located cell positions that were determined by clicking with the mouse pointer. Following the initial comparison, both the manual tracking and the automated analysis were repeated on the four identical image sets, and the algorithm was applied to identify matched objects between the replicate manual and automated operations as well. Regression analysis and t-Test calculations were performed using R Project for Statistics [V. 2.0, see ].

### Cell numbers and growth rates

Individual cells within colonies and cell clusters are not reliably recognized by current automated software using brightfield imaging. Instead, colonies and clusters are segmented as individual objects, and the areas of these objects provide a basis for estimating cell numbers. Doubling rates are calculated using linear regression of log transformed area-based cell numbers over time. Briefly, the slope of Log_2_(Cell Number) versus Time equals the doubling rate. As shown in this study, however, cell numbers do not correlate linearly will total cell area, and so doubling rates based upon an exponential growth model for area were called "area-doubling" rates.

### Experimental design, normalization, and statistical analysis

A randomized complete block design [31] was used so that all informative factors (cell-line, compound, and dose) could be individually evaluated and separated from nuisance factors contributed by between-experiment variation and technical variability as evaluated between replicate wells within each experiment.

The behaviour of the cell velocities over time was analysed using 4-hour intervals and linear regression. First, velocity measurements were averaged for wells with the same cell line and treatment. Each data-point represents the average for 8 images (acquired every 30 minutes over 4-hour time intervals) from triplicate wells in each of 3 experiments. Second, a linear trend line was fitted to each profile. Finally the intercept (initial average velocity) and the slope (behaviour of average velocity over time) were compared across cell-line and treatment. The model fitting was done using SAS Proc Mixed. The significance of coefficients in these models is tested with student's t test. Overall tests for the equivalence of starting velocities are performed using the Chi-square test. Tests for equivalence of slopes are also performed. Pair-wise comparison with Bonferroni adjustment is also employed to see differences within each pair of treatments.

The dose-response of the cell-velocities was analysed within a 24-hour period. There was considerable variation between experiments as measured by the intra-class correlation (ICC), or ratio of variance from individual factors to the total variance [32]. Therefore, prior to combining across experiments, all data for velocity and growth rates were normalized using an additive model based upon the difference between mock-coated plastic wells for each experiment. That is, the normalized measurements, Mn, were calculated from the original measurements, Mi, as follows: Mn = (Mi - Pi) + Pe, where Pe equalled the overall mean measurement for mock-coated wells for all experiments, and Pi equalled the individual means for mock-coated wells within experiments. Means were estimated using Tukey's biweight single-step M-estimator [33]. The ICC for informative factors increased from 0.41 to 0.89, while that for the major nuisance factor, between-experiment variability, decreased from 0.58 to 0.09. The proportional contribution from technical variability (replication error) increased slightly from 0.005 to 0.011 due the decrease in overall variance after normalization.

## Authors' contributions

AB assisted with design and implementation of the experiments, analysis of data and drafting of the manuscript. CA performed statistical analysis, assisted with data analysis and normalization, and contributed to software. DK designed and built the prototype as well as contributed to the software control, data analysis, and drafting of the manuscript. LQ contributed to design and description of the segmentation and tracking algorithms, construction of video examples, and custom software devices for data validation. TS designed algorithms for object matching (manual vs automated tracking) and queries for extraction of data. HW performed and summarized statistical analysis of the kinetic data. DS and YH carried out cell culture and monitoring of the automated system. JG and TC participated in design of the study and monitoring of the system. RH and LC supervised and coordinated the study. All authors read and approved the final manuscript.

## Supplementary Material

Additional File 1 **Time-lapse motility and proliferation of KM101 and MG-63 cells**. Images from four wells were combined to show the differential effects of laminin (upper left), collagen type I (upper right), collagen type IV (lower left) and mock-coated plastic (lower right). Red outlines indicate the perimeters of the cell "objects" from which object areas and centroid positions are derived. Green lines indicate tracks that are established between successive centroid positions that define the individual cell paths. In this image sequence, track segments are erased following 10 hours elapsed time. The total elapsed time of 114 hours for each cell type shows cells from shortly after seeding to near confluence. Images were acquired at 30-minute intervals and are displayed in the video at a rate of 6 images per second. Click here for file

Additional File 2 **Acceleration of MG-63 cells**. Time-lapse videos show MG-63 cells on laminin-coated (10 ug/ml, first image sequence "I04") and mock-coated plastic (second image sequence "J03"). Images were acquired at 30-minute intervals and are displayed in the video at a rate of 6 images per second. Green lines indicate automatically generated tracks connecting successive centroid positions of objects. Track segments are erased after 15 images, so the maximum track length represents 7.5 hours' migration. A total of 118 hours elapsed time is shown for each image sequence. Click here for file

Additional File 3 **Tracking scenarios**. This tracking example sequence was taken from the set of images that are shown in the upper left panel of Movie 1 in order to illustrate in more detail how the tracking algorithm handles "scenarios" such as the colliding and splitting of cells. A total elapsed time of 35 hours (70 scans) is depicted, and specific details are given in the video to explain example scenarios. Original image quality is not preserved in the videos due to necessary compression. Click here for file
